# Transcriptome analysis and RNA interference of cockroach phototransduction indicate three opsins and suggest a major role for TRPL channels

**DOI:** 10.3389/fphys.2015.00207

**Published:** 2015-07-24

**Authors:** Andrew S. French, Shannon Meisner, Hongxia Liu, Matti Weckström, Päivi H. Torkkeli

**Affiliations:** ^1^Department of Physiology and Biophysics, Dalhousie UniversityHalifax, NS, Canada; ^2^Department of Biophysics, Research Centre for Molecular Materials, University of OuluOulu, Finland

**Keywords:** *Periplaneta*, phototransduction, sensory, RNAi, electroretinogram, TRP, opsin

## Abstract

Our current understanding of insect phototransduction is based on a small number of species, but insects occupy many different visual environments. We created the retinal transcriptome of a nocturnal insect, the cockroach, *Periplaneta americana* to identify proteins involved in the earliest stages of compound eye phototransduction, and test the hypothesis that different visual environments are reflected in different molecular contributions to function. We assembled five novel mRNAs: two green opsins, one UV opsin, and one each TRP and TRPL ion channel homologs. One green opsin mRNA (pGO1) was 100–1000 times more abundant than the other opsins (pGO2 and pUVO), while pTRPL mRNA was 10 times more abundant than pTRP, estimated by transcriptome analysis or quantitative PCR (qPCR). Electroretinograms were used to record photoreceptor responses. Gene-specific *in vivo* RNA interference (RNAi) was achieved by injecting long (596–708 bp) double-stranded RNA into head hemolymph, and verified by qPCR. RNAi of the most abundant green opsin reduced both green opsins by more than 97% without affecting UV opsin, and gave a maximal reduction of 75% in ERG amplitude 7 days after injection that persisted for at least 19 days. RNAi of pTRP and pTRPL genes each specifically reduced the corresponding mRNA by 90%. Electroretinogram (ERG) reduction by pTRPL RNAi was slower than for opsin, reaching 75% attenuation by 21 days, without recovery at 29 days. pTRP RNAi attenuated ERG much less; only 30% after 21 days. Combined pTRP plus pTRPL RNAi gave only weak evidence of any cooperative interactions. We conclude that silencing retinal genes by *in vivo* RNAi using long dsRNA is effective, that visible light transduction in *Periplaneta* is dominated by pGO1, and that pTRPL plays a major role in cockroach phototransduction.

## Introduction

Animal phototransduction proceeds through light absorption by rhodopsins (opsins linked to retinal molecules), which activate ion channels via G-protein coupled second messenger pathways (Fain et al., 2010). Insect and other arthropod compound eyes contain the opsin molecules in microvilli of photoreceptor cells. Rich genetic and molecular tools have made *Drosophila* compound eyes the best-understood model of insect phototransduction (Hardie and Postma, 2008). In *Drosophila*, photon absorption by rhodopsin causes photoisomerization to metarhodopsin, which activates a heterotrimeric Gq-protein, initiating a cascade leading to activation of IP_3_ and diacylglycerol. Linkages from this cascade to opening of transient receptor potential (dTRP) and TRP-like (dTRPL) ion channels that carry the receptor current are still debated, and both chemical (Chyb et al., 1999; Huang et al., 2010) and mechanical (Hardie and Franze, 2012) intermediate steps have been proposed. In *Drosophila*, dTRP and dTRPL channels are thought to carry approximately equal parts of light-activated current under physiological conditions (Reuss et al., 1997).

Although major features of *Drosophila* phototransduction may apply to other insect species, there are probably many variations to accommodate the different visual requirements of this large and diverse group of animals. However, the study of such mechanisms has been hindered by the lack of powerful methods that can be used in *Drosophila*, including deletion/inactivation mutants and targeted mutations. Here we used the American cockroach, *Periplaneta americana*, which has a very different lifestyle to *Drosophila*, including mainly terrestrial, secluded habitats, reliance on chemical and mechanical information via prominent antennae and cerci, and preference for dark or crepuscular visual environments (Cameron, 1961). Evidence already exists that the anatomy and physiology of *Periplaneta* compound eyes are adapted to dim light (Heimonen et al., 2006, 2012), and recent electrophysiological data suggested that these differences include a larger role for TRPL than TRP channels (Immonen et al., 2014).

To explore phototransduction mechanisms we created a transcriptome of *Periplaneta* retina and assembled mRNA sequences for opsins, TRP and TRPL genes (French, 2012). To investigate the roles of each protein, we used *in vivo* RNA interference (RNAi) based gene silencing to suppress translation of these genes by injecting double stranded RNA (dsRNA) into the head hemolymph. Changes in photoreceptor function were measured by an electroretinogram (ERG) assay and the quantity of targeted mRNA measured by qPCR. Our data indicate that *Periplaneta* retina contains three opsins (pGO1, pGO2, and pUVO), with one of the green opsins, pGO1, dominating vision of visible light. *Periplaneta* pTRPL mRNA was 10-fold more abundant than pTRP, and RNAi of pTRPL was much more effective in reducing ERG, supporting a more important role for pTRPL than pTRP in *Periplaneta* phototransduction.

## Materials and methods

### Animal preparation and electrophysiology

**Figure 1:**
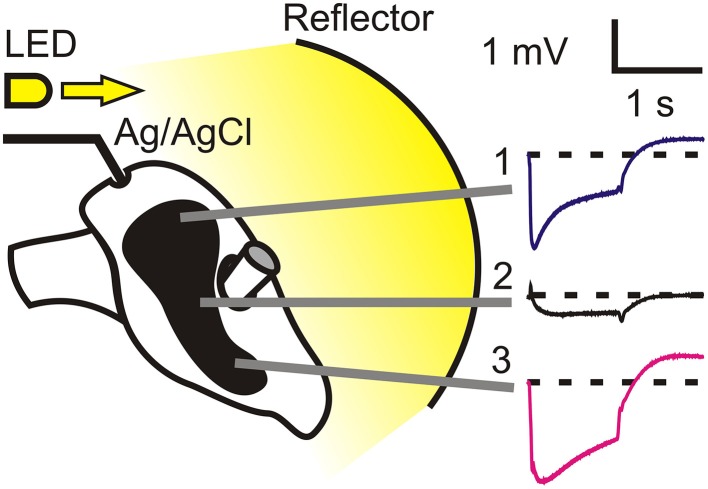
**Experimental arrangement for electroretinogram (ERG) recording**. A high intensity white light emitting diode was driven by a custom-built voltage-to-current power supply to generate 1 s flashes of 100 lm. The LED light was reflected onto the entire cockroach face by a concave white plastic reflector located 50 mm from the surfaces of the compound eyes. Sharpened tungsten electrodes were inserted just below the surface cornea, and a reference Ag/AgCl electrode through a small hole at the top of the head. ERGs varied strongly with position across the eye, as illustrated. Traces show mean and standard error responses to 10 repeated 1 s flashes. Standard errors were so small that their traces were obscured by the mean value traces.

All animal procedures followed protocols approved by the Dalhousie University Committee on Laboratory Animals. Cockroaches, *Periplaneta americana*, were raised and maintained in the laboratory at a temperature of 22 ± 2°C under a 13 h light/11 h dark cycle. Adult males were decapitated under CO_2_ anesthesia and the head mounted in a custom-built Plexiglas holder using wax as a fixative (Figure 1). Tungsten electrodes were fabricated from 0.1 mm diameter wire, sharpened electrolytically by passing current through the tip into concentrated potassium hydroxide solution, and inserted just below the corneal surface of the compound eye. A 26 g hypodermic needle was used to puncture the cuticle in the top center of the head, and a reference Ag/AgCl electrode was inserted. ERG recordings were fed to a Grass P55 amplifier (Grass Technologies, West Warwick, RI). Care was taken to ensure that severed neck trachea were open throughout the recordings.

Light stimuli were generated by a high intensity white light emitting diode (05027-PW14, LEDdynamics, Randolph, VT) driven by a custom-built voltage-to-current power supply to generate 1 s flashes of 100 lm by 1 A current. Light stimulation was directed at a concave white plastic reflector located 50 mm from the surfaces of the compound eyes. All recordings were performed within the first 3 h of the light period of the diurnal light/dark cycle. However, preliminary experiments conducted over a longer part of the cycle found no dependence of ERG on time of day. Recordings were made after 10 min of dark adaptation. Stimulation and recordings were controlled and measured by custom-written software via a personal computer and a data acquisition board (DT9812, Data Translation, Marlboro, MA). ERG signals were measured relative to the initial baseline (Figure 1) and averaged during the 1 s that the LED was illuminated.

### Retinal gene assembly

Compound eye retinas were harvested from 20 adult cockroach heads removed under CO_2_ anesthesia (40 retinas). Total RNA was extracted from the retinas using a RNeasy plus mini kit (Qiagen, Toronto, Ontario) following the manufacturer's instructions. Separation of mRNA, construction of cDNA library and Illumina processing were performed by McGill University and Génome Québec Innovation Centre, Montréal, Québec. Initial cDNA reads (89 million pairs of 100 nucleotide length) were groomed by requiring at least 80 contiguous nucleotides with Phred score >19 to give a final database of 53 million pairs of reads.

Initial sequences of interest were identified by searching the transcriptome database at low stringency vs. coding regions from published genes of other insects. Searches were performed against both nucleotide and amino-acid sequences. Amino-acid searches were conducted using BLOSUM45, BLOSUM62, and BLOSUM80 matching matrices (Henikoff and Henikoff, 1993). All putatively matching reads were compared to the non-redundant protein database using BLASTX (http://blast.ncbi.nlm.nih.gov). Identified reads of interest were extended by the transcriptome walking algorithm (French, 2012). Walking was continued to include the complete protein coding sequence (mORF or CDS) and the STOP codon in the 3′ direction.

### RT-PCR verification of assembled genes

Reverse transcription (RT) was performed using total RNA extracted from the retinas and oligo d(T)_23_VN primers with ProtoScript II reverse transcriptase (New England BioLabs, Whitby, Ontario). The synthesized cDNA was used as template to amplify different regions of the genes using a series of primer designs based on the assembled sequences. PCR product sizes were verified by running the product with a DNA marker on an agarose gel.

### RNA interference

RT-PCR was performed as above. RT product was used in PCRs to amplify the template DNAs using Q5 High-Fidelity DNA Polymerase (New England BioLabs). Primers used for the target DNA region of KC292630, KC329816, and KP861985 were tailed with the T7 promoter sequence TAATACGACTCACTATAGGG at their 5′ end (Table 1). PCR products were purified with GenElute Gel Extraction Kit (Sigma, Oakville, Ontario) and used to synthesize dsRNA with the MEGAscript RNAi Kit (Ambion, Life Technologies, Burlington, Ontario) following the manufacturer's instructions. The quality of dsRNA was confirmed by running an agarose gel and the concentration was determined by spectrophotometry.

**Table 1:** **Primers used to synthesize dsRNA templates for RNAi (T7 promoter in upper case)**.

**Gene and direction**	**Sequence**	**dsRNA size (bp)**
pGO1 forward	TAATACGACTCACTATAGGGtgggccttctctattggatg	596
pGO1 reverse	TAATACGACTCACTATAGGGggtgtcttctcctcgctgac	
pTRP forward	TAATACGACTCACTATAGGGcacagccgagactaggaagaga	692
pTRP reverse	TAATACGACTCACTATAGGGgaattgaaatgtccgaggtttc	
pTRPL forward	TAATACGACTCACTATAGGGaaagagcgtgattaccgctac	708
pTRPL reverse	TAATACGACTCACTATAGGGacgagatgcttggttgctct	

For *in vivo* RNAi, a 26 g hypodermic needle was used to create a small hole in the midline of the head cuticle between the eyes under CO_2_ anesthesia. Custom-made injection pipets were fabricated by joining the tips of borosilicate glass microelectrodes to the cut ends of 5 mL plastic transfer pipets (Fisher Scientific, Ottawa, Ontario) using wax. Double-stranded RNA (4 μg in 1 μL cockroach saline) was injected through the hole into the head tissue. Hypodermic needles and glass pipets were washed between injections with RNaseZAP (Sigma) and then rinsed with RNase-free water. Fresh needles and pipets were used for each batch of injections. After recovery from anesthesia, animals were returned to separate cages in the same animal room as the main colony, and provided with food and water *ad lib*. Control animals either received no injections or 1 μL injections of cockroach saline.

### Quantitation of mRNA expression by real-time qPCR

Total RNA was extracted from retinas of treated animals after the indicated number of days using the RNeasy Plus mini kit (Qiagen). After treatment with RNase free DNase I (Ambion), the quality and quantity of RNA were analyzed using an Experion RNA Analysis Kit (Bio-Rad, Mississauga, Ontario). Fifty nanogram of total RNA was used for first-strand cDNA synthesis using the ProtoScript II reverse transcriptase (New England BioLabs). qPCR was performed using the GoTaq® qPCR Master (Promega, Madison, WI) on a CFX96™ real-time PCR detection system (Bio-Rad), as described previously (Taylor et al., 2010). Gene expression levels and PCR efficiency, along with its standard error, were calculated using the Bio-Rad CFX Manager, version 3.1 (Bio-Rad), which employs a proven comparative Cq (ΔCq) relative quantitation model with PCR efficiency correction and multiple reference gene normalization using GAPDH and Actin. Primer sequences for the specific and reference genes are listed in Table 2. Amplification efficiencies of primers were determined using serially diluted cDNA samples (Table 2). All PCR runs were performed in triplicate and the data analyzed by CFX Manager software (Bio-Rad).

**Table 2:** **Primers used for qPCR analysis**.

**Gene and direction**	**Sequence**	**Amplicon (bp)**	**Efficiency (%)**
pGO1 forward	TATTGTACAGGCCGTCGCAG	159	87.50
pGO1 reverse	CATGCGAAGAACCACAGAGAG		
pUVO forward	GGTGTGAGCATGATTCCTGC	152	94.53
pUVO reverse	CTGGTCTCACTCTGTGTTGGTG		
pGO2 forward	CATTCCTCGTGTGTGGAGAA	97	96.26
pGO2 reverse	CTAAGCCTTTTCAGCAGCTG		
pTRP forward	CGCATAAGAAGGCTATGGACAG	124	90.07
pTRP reverse	CGAATCTCCATCACGTCGTC		
pTRPL forward	GCAGTGCTCAATTCCTCTTCAC	164	93.19
pTRPL reverse	GGAGTCGGACTGCGGTTAAC		
Actin forward	GTACGTTGCTATCCAGGCTGTG	158	85.60
Actin reverse	AATCGCGACCAGCCAGATC		
GAPDH forward	GTGTTCCTGTTCCCAATGTTTC	134	89.51
GAPDH reverse	TTCAGTGTAGTCCAAGATGCC		

### Molecular phylogenetic analysis

Evolutionary history for arthropod opsins and TRP channels were inferred by maximum likelihood. The TRP homologs were identified by BLAST analysis of non-redundant arthropod protein sequences from GenBank and UniProt using the *Periplaneta* TRP and TRPL as queries. We added the three new opsins found in *Periplaneta* retina to those used in a previous phylogenetic analysis for cricket opsins (Henze et al., 2012). Sequences were aligned by an online version of multiple sequence alignment software MAFFT Version 7 (http://mafft.cbrc.jp/alignment/server/) with default parameters (BLOSUM62, gap opening penalty 1.53) and G-Ins-I strategy with 50 SwissProt homologs and threshold *E* = 1e-10 (Katoh and Standley, 2013). Maximum likelihood analysis was performed with MEGA 6 software (Tamura et al., 2013). Based on the results from MEGA 6 “Models” the best protein model for the MAFFT aligned TRP sequences was a Gamma distributed Le-Gascuel model (LG + G). For opsins the Le-Gascuel model with frequencies (LG + G + F) was used (Le and Gascuel, 2008). Both analyses were performed with 1000 bootstraps using subtree pruning and extensive regrafting options with other settings at the defaults. The initial trees for the heuristic searches were obtained by applying the neighbor-joining method to a matrix of pairwise distances estimated using a JTT model. The TRP analysis involved 27 amino acid and the opsin analysis 48 amino acid sequences. All positions with less than 95% site coverage were eliminated. That is, fewer than 5% alignment gaps, missing data, and ambiguous bases were allowed at any position.

### Statistical inference

Mean ERG amplitudes from untreated and treated different animals were tested by the Mann–Whitney nonparametric two-tailed test for the significance of difference between distributions of two independent samples of data that are not normally distributed. Statistical significance in the figures is indicated by asterisks: ^*^p < 0.05, ^**^p < 0.01, ^***^p < 0.001.

## Results

### pGO1 was the most abundant opsin and pTRPL the most abundant TRP gene

Exhaustive, low-stringency, searches of the transcriptome library were conducted for opsins and TRP-family ion channels, using published amino-acid sequences for similar genes in several insect species, including particularly: *Drosophila melanogaster, Gryllus bimaculatus*, and *Tribolium castaneum*. On the basis of sequence similarity two genes were identified as green opsins: pGO1 (GenBank KP861985) and pGO2 (KP981367). A third gene was identified as UV-sensitive opsin, pUVO (KP941115). Extensive searches were conducted for a blue opsin, particularly using the Gryllus blue opsin as a template, but these failed to find any matching reads. Searches for TRP-family genes found two sequences identified as pTRP (KC329816) and pTRPL (KC292630) ion channels.

**Figure 2:**
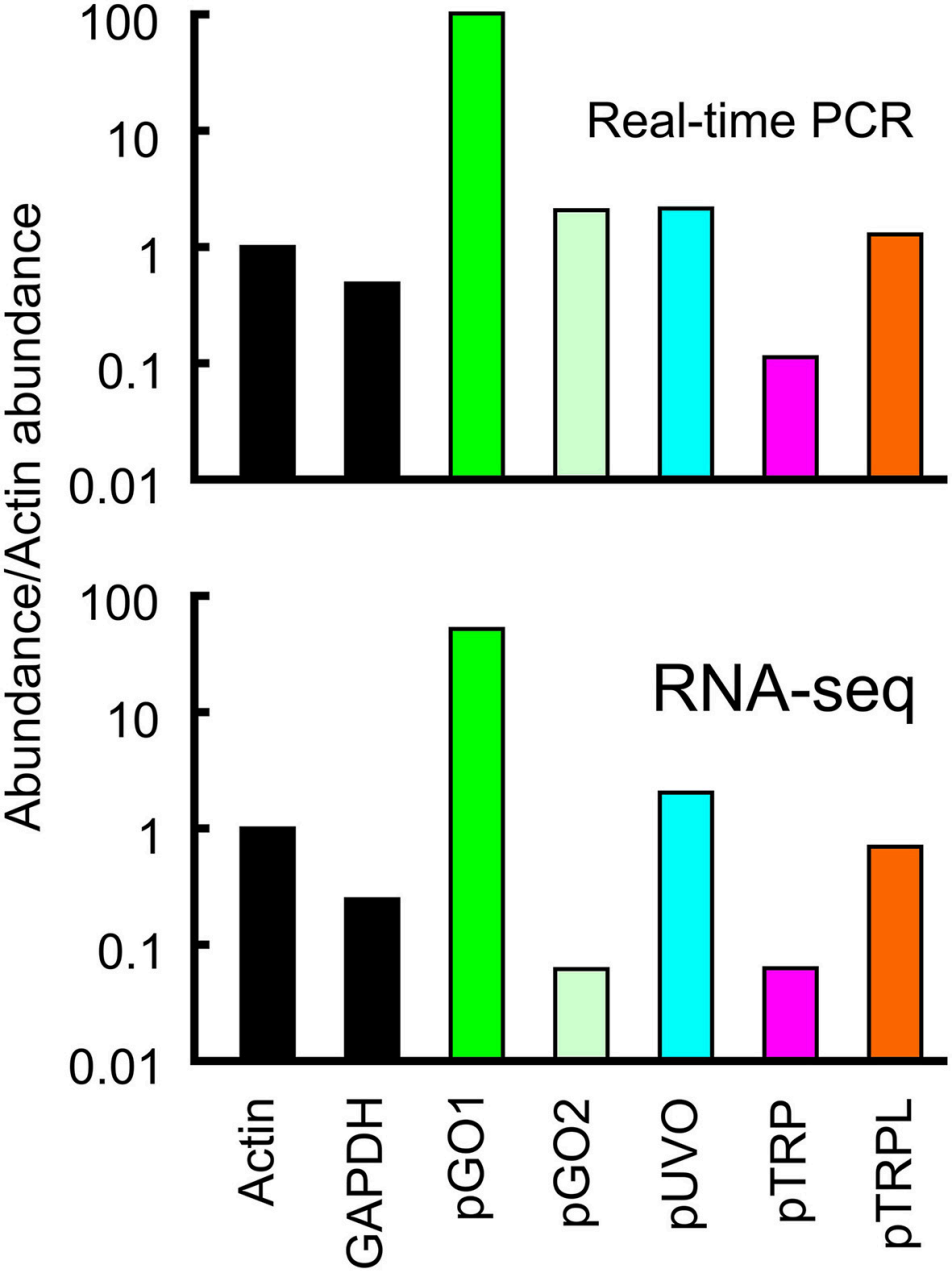
**Relative abundances of mRNA sequences in total retinal RNA**. Data values were normalized to the concentration of actin. The relative abundance of a second housekeeping gene, GAPDH, is also shown for comparison. Upper panel data were obtained from qPCR. Lower panel data were obtained by counting total reads in the transcriptome library with at least 90 consecutive identical nucleotides to the reading frame of the gene, then dividing by the reading frame length. Note the agreement between data from the two estimation methods. Both abundance scales are logarithmic. Green opsins, pGO1, pGO2; UV opsin, pUVO; TRP channels, pTRP and pTRPL.

Relative abundance of transcribed mRNA in the retina was estimated by two methods. First, by searching the complete groomed transcriptome library for reads matching the reading frame of each gene, using the criterion of at least 90/100 identical nucleotide matches to score each read as derived from the gene. Total counts were normalized for gene length, and expressed as abundance relative to the putative actin gene. Second, by real-time quantitative PCR (qPCR) of retinal RNA using primers based on the identified genes, and comparing cycle numbers to reach the quantification threshold, Cq, again relative to the actin gene. Relative abundances of mRNAs agreed well for the two methods (Figure 2). For the opsins, pGO1 was the most abundant at ~100 times more than pGO2 and 25–100 times more than pUVO. pTRPL mRNA was ~10 times more abundant than pTRP by both methods.

### Phylogeny of cockroach opsins and TRP channels within the arthropods

**Figure 3:**
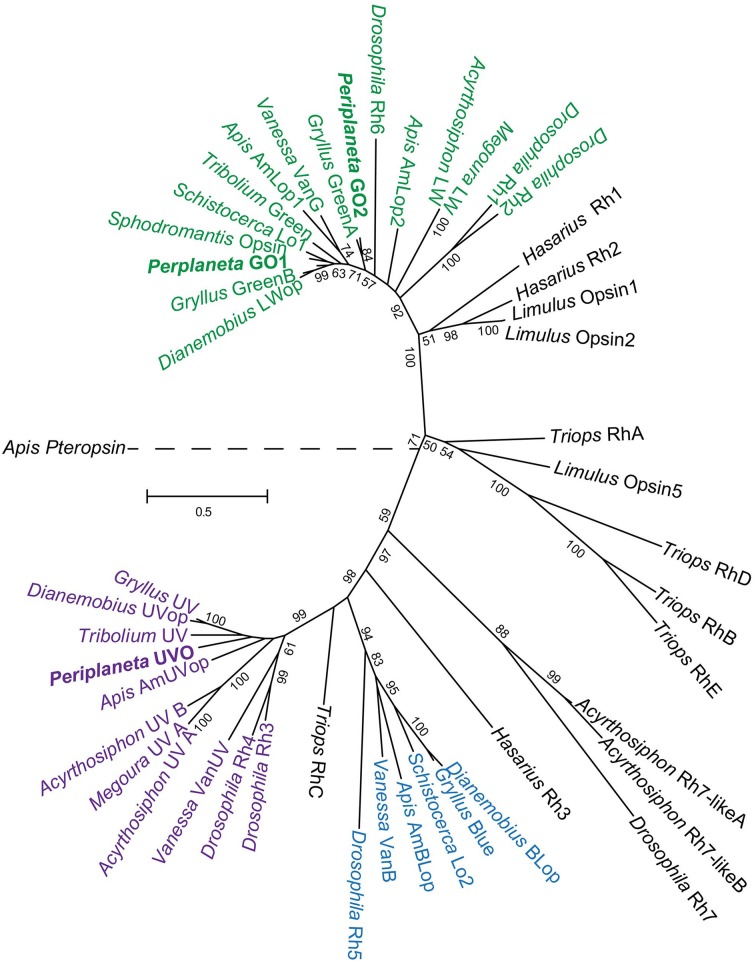
**Molecular phylogenetic analysis of arthropod opsins by maximum likelihood method**. The tree is based on MAFFT aligned full-length sequences with honeybee pteropsin as an outgroup. Bootstrap values are indicated for all nodes supported at more than 50%. The tree is drawn to scale, with branch lengths measured in numbers of substitutions per site. Opsins are colored based on optimum wavelengths: purple for ultraviolet, blue, and green for their respective clades. Accession numbers for genes used: *Vanessa cardui* VanG, AY613986; VanB, AY613987; VanUV, AF414074; *Drosophila melanogaster* Rh1, K02315; Rh2, M12896; Rh3, M17718; Rh4, M17730; Rh5, U67905; Rh6, Z86118; Rh7, NM_079311; *Apis mellifera* AmLop1, U26026; AmLop2, BK005515; AmBLop, AF004168; AmUVop, AF004169; Pteropsin, BK005510; *Tribolium castaneum* Green, M_001162519; UV, XM_965251; *Megoura viciae* LW, AF189714; UV_A, AF1897145; *Schistocerca gregaria* Lo1, X80071; Lo2, X80072; *Gryllus bimaculatus* Green A, HM363620; Green B, HM363621; Blue, HM363622; UV, HM363623; *Dianemobius nigrofasciatus* LWop, FJ232921; BLop, AB291232; UVop, AB458852; *Sphodromantis* spec. Opsin, X71665; *Triops granaries* RhA, AB293428; RhB, AB293429; RhC, AB293430; RhD, AB293431; RhE, AB293432; *Hasarius adansoni* Rh1, AB251846; Rh2, AB251847; Rh3, AB251848; *Limulus polyphemus* Opsin1, L03781; Opsin2, L03782; Opsin5, FJ791252.

*Periplaneta* is a member of the Blattodea order of insects, but little information is available about opsin or TRP genes of this order. Molecular phylogenetic analysis (Figure 3) placed the two *Periplaneta* green opsins closest to orthopteran insects, with pGO1 and pGO2 closest to cricket (*Gryllus bimaculatus*) Green B and Green A, respectively. The UV opsin was close to orthopterans, including *Gryllus*, but also to the coleopteran flour beetle (*Tribolium castaneum*). The close relationships to *Gryllus* are of interest because expressions of cricket opsins have been localized within its compound eyes and ocelli (Henze et al., 2012).

**Figure 4:**
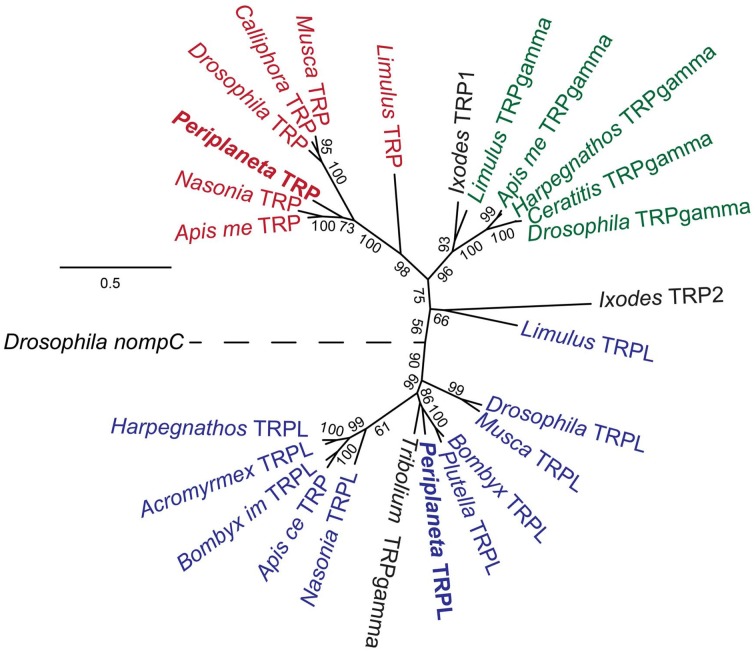
**Molecular Phylogenetic analysis of arthropod TRPC channels by Maximum Likelihood method**. The tree is based on MAFFT aligned full-length sequences with *Drosophila* TRPN channel nompC as an outgroup. Bootstrap values are indicated for all nodes. The tree is drawn to scale, with branch lengths measured in the number of substitutions per site. TRP channels are shown in red, TRPgamma channels in green, and TRPL channels in blue. Channels that have not been functionally classified in GeneBank, or may have incorrect classification, are shown in black. Accession numbers for genes used: *Acromyrmex echinatior* TRPL, F4X8T0; *Apis cerana* TRP, V9IJ69; *Apis mellifera* TRP, A0A087ZR90; TRPgamma, A0A088AV64; *Bombyx impatients* TRPL, XP_003490000; *Bombyx mori* TRPL, XP_004922702; *Drosophila melanogaster* nompC, Q7KTN8; TRP, P19334; TRPgamma, Q9VJJ7; TRPL, P48994; *Calliphora vicina* TRP, Q94447; *Ceratitis capitata* TRPgamma, W8BIE6; *Harpegnathos saltator* TRPgamma, E2BGT4; *Ixodes scapularis* TRP1, B7PBX3; TRP2, B7Q558; *Musca domestica* TRP, T1P894; TRPL, T1PFK2; *Limulus polyphemus* TRP, A0A059XJT4; TRPgamma, A0A059XPJ5; TRPL, A0A059XPN0; *Nasonia vitripennis* TRP, K7J2P6; TRPL, K7IZ16; *Plutella xylostella* TRPL, XP_011560938; *Tribolium castaneum* TRPgamma, D2A1G1.

The invertebrate TRP channels are members of the TRPC superfamily (Hardie, 2014). Molecular phylogenetic analysis (Figure 4) placed pTRP in the same clade with insect TRP channels, closer to the hymenopteran than dipteran order of insects. The pTRPL channel was in the same clade with other TRPL channels, closest to red flour beetle, *Tribolium castaneum*, channel that was annotated in GenBank as TRPgamma, but clearly falls into the TRPL clade. Another *Tribolium* sequence has been assigned as TRPL (Accession number D2A2N4), but was phylogenetically distant to any of the TRP sequences in our analysis. We have not found any TRPgamma sequences in the *Periplantea* retina transcriptome.

### RNA interference specifically reduced mRNA levels in the retina

We initially used short dsRNA in attempts to induce RNAi (Elbashir et al., 2001). Dicer-substrate dsRNA sequences of 27 nucleotides with overhanging DNA nucleotides were designed using standard algorithms, constructed commercially and injected into the head hemolymph, or directly into one eye. Injections of short RNA were used alone, and combined with a wide variety of commercially available transfection agents, over a range of concentrations and time frames. However, no consistent RNAi was seen with any attempt. In contrast, long (500–700 bp) dsRNA (Miller et al., 2012) was highly effective in reducing mRNA concentration and attenuating the ERG, so this method was used in all further experiments.

**Figure 5:**
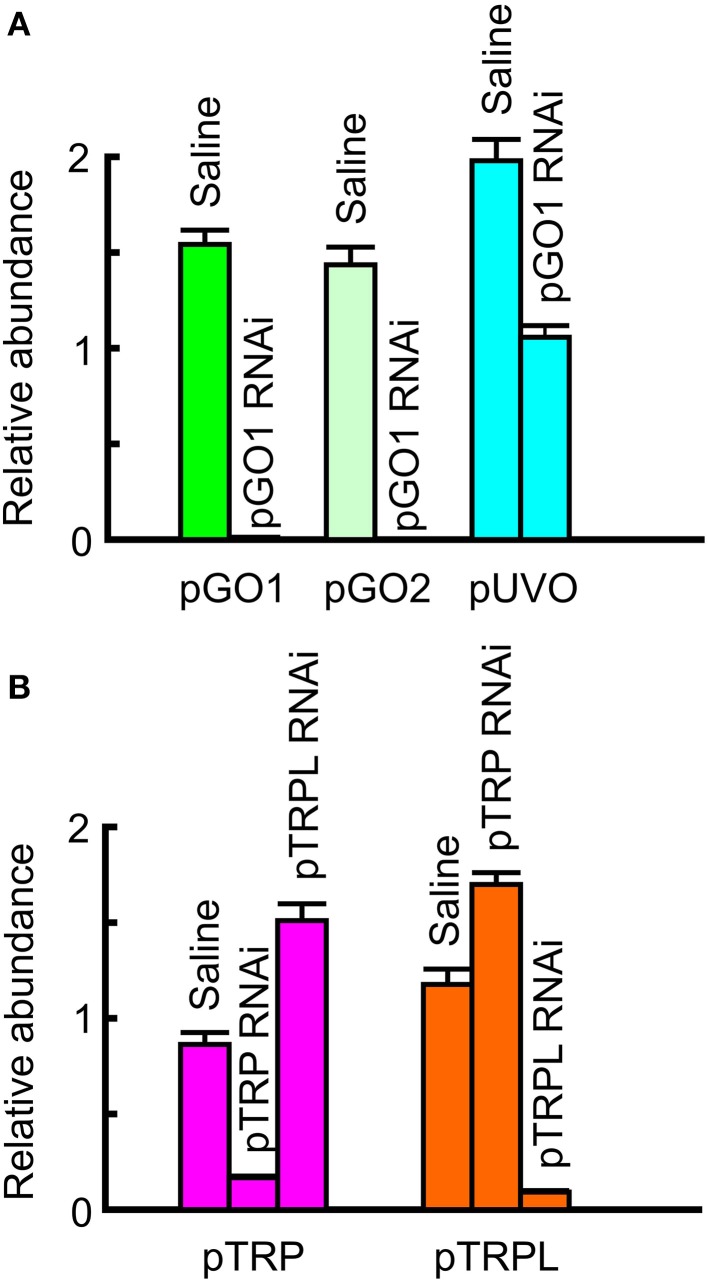
**Reduction in mRNA concentration by RNA interference (RNAi)**. Data were obtained by qPCR, using the mean of actin and GADPH mRNA abundances as reference, and compared to untreated controls. **(A)** RNAi by green opsin 1 (pGO1) gene reduced pGO1 to 0.012 of control and green opsin 2 (pGO2) to 0.001 of control. UV opsin (pUVO) concentration was 1.06 of control. **(B)** RNAi by pTRP channel gene reduced pTRP to 0.17 of control, while pTRPL was 1.51 of control. RNAi by pTRPL gene reduced pTRPL to 0.09 of control, while pTRP was 1.7 of control. All values are mean ± sd, *n* = 3.

RNAi was performed by injecting dsRNA that targeted the highly abundant pGO1 opsin as well as pTRP and pTRPL. Levels of retinal mRNA were estimated by qPCR 21 days after dsRNA injections. Each measurement used eight injected animals, giving 14 or 16 retinas, depending on survival. Result from injected animals were compared to uninjected control animals (relative abundance = 1) and saline injected animals (Figure 5). Levels of both pGO1 and pGO2 opsins were profoundly reduced by pGO1 RNAi (0.012 and 0.0013 of control respectively) while the abundance of pUVO was unaffected. Cockroach saline sham injections sometimes increased mRNA levels by 50–100%, but not consistently. Levels of pTRP and pTRPL mRNA were also specifically reduced by their respective RNAi. pTRP mRNA was reduced to 0.16 of control, and pTRPL mRNA was reduced to 0.09 of control. In both cases, levels of mRNA were actually increased about 50% by injection of the alternate dsRNA. Saline injections again had no effect, or increased mRNA levels. Combined RNAi of pTRP and pTRPL together reduced both mRNA levels after 7 days (pTRP 0.084, pTRPL 0.0630) and 16 days (pTRP 0.057, pTRPL 0.028).

### ERG recordings varied with retinal region

To test the effects of RNAi on vision, we made ERG recordings using 1 s duration flashes of white light. The flashes were repeated at 10 s intervals and the average response was recorded. The cockroach eye has an irregular shape that deviates and narrows as it passes around the prominent antennae (Figure 1). ERG signals varied strongly in form and amplitude with location across the eye, giving transient traces at the top of the eye, very small amplitude signals in the center, and larger, less transient responses at the bottom of the eye. We named these three recording locations Positions 1–3, as indicated in Figure 1. All further experiments used only Positions 1 and 3. Recordings from control animals were highly reliable. The traces shown in Figure 1 are mean ± standard error values from 15 animals (30 retinas), but the error values are obscured by the mean values. No significant differences were ever seen between the responses of the left and right eyes, so data from both eyes were pooled.

### Green opsin RNAi strongly reduced ERG amplitude

**Figure 6:**
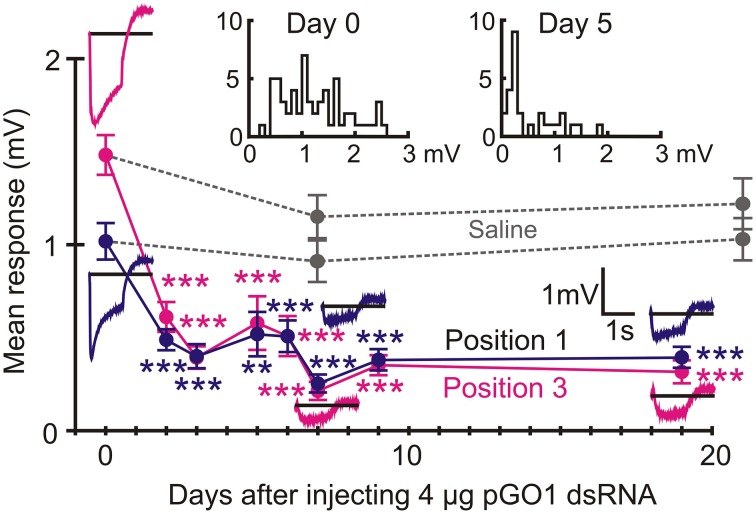
**RNAi by pGO1 reduced ERG significantly after 2 days**. Data (mean ± se) are shown for ERG recordings from Positions 1 to 3 (Figure 1) at different times after injection with 4 μg dsRNA. Raw traces show example recordings. Data points are mean trace values during the 1 s flash of light. Saline injections gave no reductions in ERG. Histograms (upper) show all mean ERG values (combined Positions 1 and 3) at days 0 and 5 of RNAi. Note that a small number of animals always continued to give ERG values in the normal range. Numbers of recordings were 30 for controls and 14 or 16 for RNAi or saline treatments. Asterisks: ^**^p < 0.01, ^***^p < 0.001.

ERG recordings from Positions 1 and 3 were measured from groups of animals injected with pGO1 dsRNA for periods ranging from 2 to 19 days after injection (Figure 6). Each recording day had initially eight animals, but in a few cases one died prematurely, so each treatment data point in the figure resulted from 14 or 16 separate retinal recordings (two eyes per animal) in each recording position. Mean ERG amplitude during the time of the light flash was measured. ERG amplitude was significantly reduced 2 days after injection, maximum attenuation was reached after 7 days, and persisted for at least 19 days (Figure 6). Variability of mean amplitude between days of treatment was largely caused by differences in the extent of RNAi seen in different animals. This is illustrated by two inset histograms in Figure 6 that show distributions of responses in 15 control animals and eight animals injected 5 days previously. While the distribution shifted strongly to lower amplitudes after RNAi, there were often one and in some cases up to three animals with responses in the normal range. Data from different sets of animals with sham saline injections were tested after 7 days (time for peak reduction by pGO1) and 21 days. ERG amplitudes from saline injected animals were not significantly different to control animals (Figure 6).

### RNAi of pTRP channels reduced ERG much less than pTRPL channels

**Figure 7:**
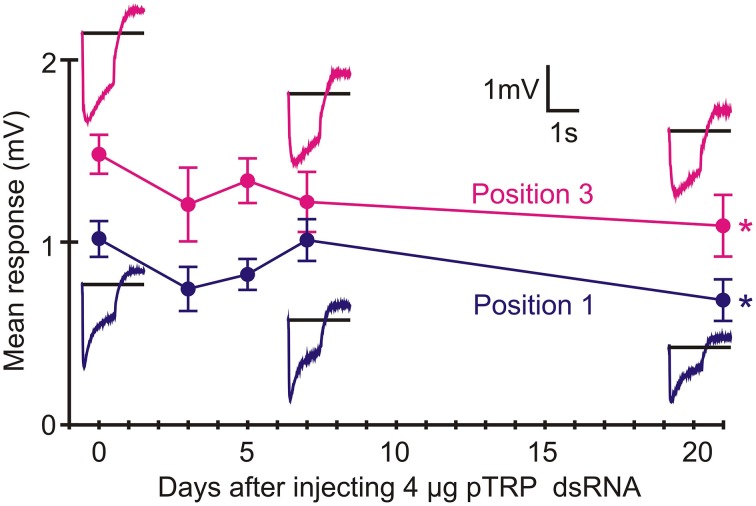
**RNAi of pTRP reduced ERG after 21 days**. Data presentation similar to Figure 6. Only marginally significant reductions in means values were seen after 21 days (Position 1: *p* = 0.022, Position 3: *p* = 0.031). Numbers of recordings were 30 for controls and 14 or 16 for RNAi treatments. Asterisks: ^*^p < 0.05.

**Figure 8:**
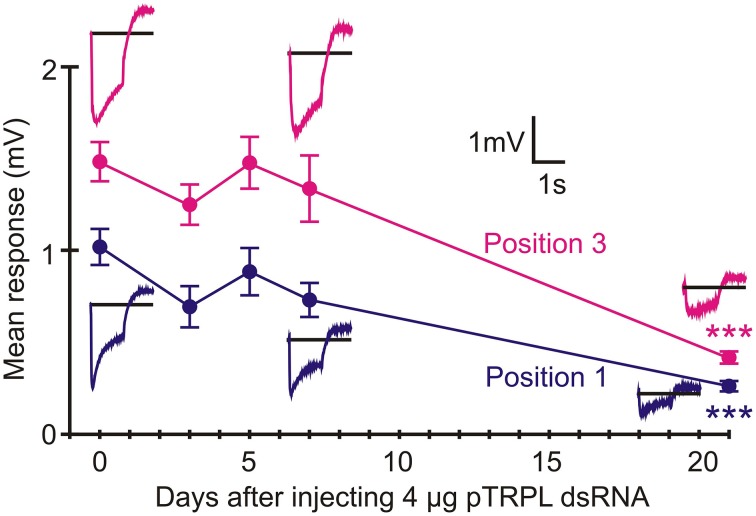
**RNAi of pTRPL reduced ERG strongly after 21 days**. Data presentation similar to Figure 6. Statistical significance of mean reduction for both positions after 21 days was *p* < 0.0001. Numbers of recordings were 30 for controls and 14 or 16 for RNAi treatments. Asterisks: ^***^p < 0.001.

Twenty-one days after injection with pTRP channel dsRNA, ERGs were only weakly reduced (Position 1: 67% response *p* = 0.022, Position 3: 74% response *p* = 0.031, Figure 7). In contrast, RNAi of the pTRPL channel gene produced similar attenuation to green opsin RNAi (about 75% reduction, *p* < 0.0001), although the effect was much slower, becoming strongly significant after 21 days (Figure 8). The effect of pTRPL RNAi persisted at the same level after 29 days (data not shown).

**Figure 9:**
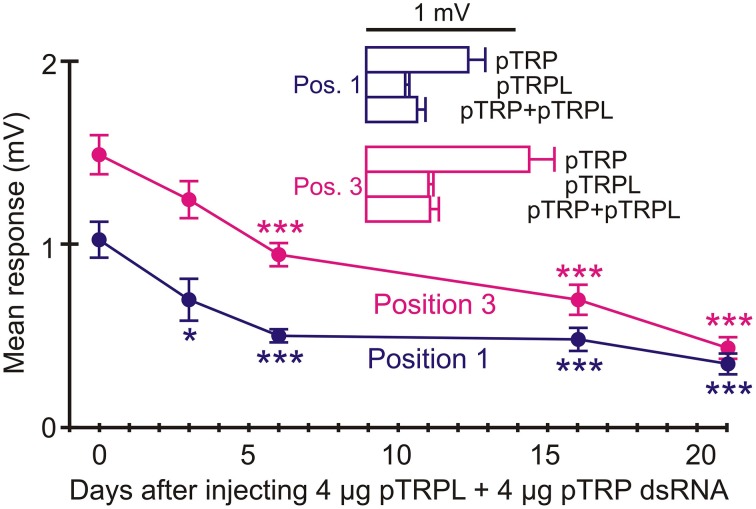
**RNAi of combined pTRP and pTRPL reduced ERG significantly after 6 days**. Animals received injections containing 4 μg pTRP and 4 μg pTRPL dsRNA. Significant reduction in ERG was seen after 6 days and persisted at 21 days. Upper histograms show mean values at both positions after 21 days treatment with pTRP, pTRPL, and pTRP+pTRPL RNAi. Note that the mean values for pTRPL alone and combined injections were not significantly different. Numbers of recordings were 30 for controls and 14 or 16 for RNAi treatments. Asterisks: ^*^p < 0.05, ^***^p < 0.001.

Possible interactions between pTRP and pTRPL channels were tested by injecting animals with dsRNA for both pTRP and pTRPL (Figure 9). Significant attenuation of the ERG signal was now seen after 6 days, but only reached the same level as pTRPL alone, of 75% reduction, after 21 days. Inset histograms (Figure 9) compare the maximum attenuations seen for TRP, TRPL, and the combined injections for both recording positions. There was no evidence for any greater or less attenuation for the combination than seen with pTRPL alone.

## Discussion

### *In vivo* insect RNAi

RNAi in living insects is becoming an important technology for controlling medically and agriculturally important pests, in addition to its utility as a research tool. A range of delivery methods have been used, including direct injection into body cavities to induce systemic RNAi (Wynant et al., 2014). However, the effectiveness of direct injection varies strongly with the species, tissue, and gene involved (Belles, 2010; Wynant et al., 2014). A systematic study of RNAi by injection in the larval red flour beetle, *Tribolium castaneum*, found that effectiveness increased strongly with dsRNA length, and implicated cellular uptake as a major limiting factor (Miller et al., 2012). Our results support these conclusions and show that long dsRNA produces robust and long lasting RNAi also in an adult animal. The effectiveness of uptake in the case of photoreceptors may be based on their active membrane turnover mechanisms, involving pinocytosis (Blest, 1988). While the methods that we used produced effective RNAi, we observed that one or more animals in each treated batch of eight gave normal ERG recordings, with both retinas apparently unaffected (Figure 6). This suggests that either our injection method was sometimes ineffective, or that some animals were able to mount a systemic defense against the dsRNA injection. Another possible limitation of RNAi is the possibility of cross-reactivity causing suppression of genes other than the target. While this cannot be eliminated, the data that we observed for green vs. UV opsins, and TRP vs. TRPL channels indicates that the effects we measured were specific to the targeted genes in each case.

### Cockroach opsins

The opsin family of genes is large and complex (Porter et al., 2012). Insect compound eye opsins are members of the Gq subgroup of microvillar opsins, with variable numbers in different species. An earlier attempt to reduce opsin concentration in the bee compound eye by injecting 267 bp long dsRNA, gave only transient reduction of mRNA concentration without any effect on ERG (Leboulle et al., 2013). No descriptions of opsin genes in other cockroaches are available, but a detailed examination of opsins in the cricket, *Gryllus bimaculatus*, found four opsins characterized as UV, Blue, Green A, and Green B (Henze et al., 2012). Only the UV, Blue, and Green B genes were found in the compound eyes, with Green A in the simpler ocelli. Our *Periplaneta* sequences were all obtained from compound eyes, and the two longer wavelength genes, pGO1 and pGO2 closely aligned to cricket Green B and Green A, respectively (Figure 3).

Cricket opsins were differentially expressed in separate regions of the compound eye. Green B was strongly expressed in all regions except for the dorsal rim, which is specialized for sky vision, and had mainly Blue opsin. No relative abundance values were given for the different mRNA molecules in the entire cricket compound eye, but the strong expression of Green B in most of the eye (Henze et al., 2012) agrees with our finding that the dominant pGO1 of *Periplaneta* (Figure 2) is closest to Green B by molecular phylogenetic analysis (Figure 3). Our finding of pGO2 in the compound eye differs from the finding that Green A was only present in cricket ocelli, but homologous green opsins from other insects in the same group are present in compound eyes (Henze et al., 2012). Absence of a blue opsin was unexpected, especially because the green and UV opsins were strongly represented in the transcriptome data, and other genes, such as potassium channels were easily assembled from abundances almost a 100 times lower than the opsins. It may indicate complete loss of the gene, or at least its expression.

Electrophysiology of *Periplaneta* compound eyes gave two classes of photoreceptors with spectral sensitivity peaks in the UV and green at 365 and 507 nm (Mote and Goldsmith, 1970) with uniform distribution of the two types across the eye (Butler, 1971). Lack of blue-sensitive cells or opsins in *Periplaneta* correlates with the absence of a specialized dorsal rim area, but the relative roles of the two green opsins are unknown. Since the mRNA concentrations of both green opsins were similarly reduced by RNAi, the present data cannot separate their contributions. However, the profound reduction in white light stimulated ERG by pGO1 RNAi indicates that the two green opsins dominate visible light transduction in the *Periplaneta* compound eye, so contributions from other, unknown long wavelength opsins are unlikely.

### The *Periplaneta* electroretinogram

ERG recordings from compound eyes are generally difficult to relate to individual cellular components because of the complex tissue structure that includes electrical barriers and current sinks (Shaw, 1975). However, although insect ERG can contain contributions from cells other than photoreceptors, this is unlikely in the cockroach, because the other visual ganglia are located very far proximally (Ribi, 1977; Mote, 1990). ERG recordings from another cockroach species, *Blattella germanica*, had similar amplitude and polarity, although detailed comparison is difficult because much shorter flashes were used (Chang and Lee, 2001). The strong variation that we observed with position across the eye has not been observed before, and recording position was not described in the *Blattella* data. It seems likely that the irregular shape of the *Periplaneta* compound eye complicates the electrical current paths flowing through the eye, leading to the dependence of amplitude, and waveform on position that we observed. Although this positional variation in ERG is interesting and merits further investigation, it did not affect our RNAi data because both recording positions showed similar attenuation.

### Which ion channels carry the light-activated current in *Periplaneta*?

In *Drosophila* compound eye photoreceptors, light is detected by rhodopsin molecules, and leads to opening of two types of ion channels, dTRP and TRP-Like (dTRPL) (Hardie and Minke, 1992; Hardie and Raghu, 2001). However, the final step in the phototransduction leading to ion channel activation is not yet clear (Hardie and Franze, 2012; Hardie and Juusola, 2015). The ionic selectivities of the two ion channels in *Drosophila* are different, with dTRP more Ca^2+^-selective than dTRPL. Relative contributions of the two channels to total light-activated current were proposed to be approximately equal under physiological conditions, based on noise analysis and reversal potentials with bi-ionic conditions (Reuss et al., 1997). Using whole-cell light-activated current recordings from isolated *Periplaneta* photoreceptors, Immonen et al. (2014) found significantly less dependence of reversal potential on calcium concentration, and suggested that a larger fraction of current is carried by pTRPL channels in *Periplaneta* compared to *Drosophila*. These two species have very different lifestyles. The fruit fly is dependent on rapid aerial visual function under bright daylight, whereas the cockroach is mainly terrestrial, living under dark, or dim conditions. Such profound differences in visual ecology are known to affect how visual system functions, including mechanisms mediated by various ion channels (Laughlin and Weckström, 1993; Weckström and Laughlin, 1995). In this sense, differences in contributions of TRP and TRPL channels to phototransduction in the two species are not surprising, although unexpected.

The present data support a larger role for pTRPL than pTRP channels in *Periplaneta* compound eye phototransduction. pTRPL mRNA was 10 times more abundant than pTRP, suggesting a higher concentration of the protein in photoreceptors. RNAi effectively and selectively reduced either pTRPL or pTRP mRNA concentrations (Figure 5), yet pTRPL reduction was highly effective in reducing ERG (Figure 8), whereas pTRP reduction had only a marginal effect on ERG (Figure 7). The qPCR results suggest that silencing the pTRPL gene upregulated pTRP and vice versa (Figure 5), which may indicate a compensatory mechanisms regulating the number of transduction channels, similarly to the case of voltage-gated K^+^ channels in *Drosophila* (Niven et al., 2003; Vähäsöyrinki et al., 2006). While increased pTRPL in animals with silenced pTRP may have contributed to maintenance of their visual function (Figure 7), any increased pTRP did not allow pTRPL knockdowns to preserve their ERG responses (Figure 8). Combined suppression of both pTRP and pTRPL caused a more rapid reduction in ERG (Figure 9), suggesting some effect of pTRP channels on phototransduction, but the final ERG reduction was indistinguishable from pTRPL reduction alone, indicating a major role for TRPL in carrying the light-activated current.

The fact that we did not find a TRPgamma sequence in the *Periplaneta* retina transcriptome suggests that this channel is not important in cockroach vision. Its significance in phototransduction is also doubtful in *Drosophila*, where TRPgamma was shown to be a Ca^2+^ permeable non-selective cation channel that is expressed in many body regions, and no special accumulation in the eye (Jörs et al., 2006).

## Conclusions

Visible light detection by *Periplaneta* compound eyes utilizes two green opsins, although their relative roles are not yet clear. One opsin, pGO1, appears dominant, and is structurally close to the dominant opsin in cricket compound eyes. Only two types of TRP ion channels were found, corresponding structurally to the *Drosophila* dTRP and dTRPL channels. However, unlike *Drosophila*, the relative amounts and functional contributions of the two channels were dissimilar, with more pTRPL in the eye and a larger reduction in ERG caused by RNAi of pTRPL.

*In vivo* RNAi with long dsRNA provides an effective method of selectively reducing gene expression in insect tissues, including compound eye photoreceptors. Combination of this approach with more detailed electrophysiological and pharmacological methods offers promise in elucidating phototransduction mechanisms in species where earlier molecular and genetic methods, such as mutant analysis or targeted mutations, cannot be used, as well as in exploring the molecular basis of adaptation to different visual environments.

### Conflict of interest statement

The authors declare that the research was conducted in the absence of any commercial or financial relationships that could be construed as a potential conflict of interest.
